# Melatonin Improves Vasogenic Edema via Inhibition to Water Channel Aquaporin-4 (AQP4) and Metalloproteinase-9 (MMP-9) Following Permanent Focal Cerebral Ischemia

**DOI:** 10.3390/biomedicines12102184

**Published:** 2024-09-26

**Authors:** Ai-Hua Lee, Shih-Huang Tai, Sheng-Yang Huang, Li-Der Chang, Liang-Yi Chen, Yu-Ning Chen, Hao-Hsiang Hsu, E-Jian Lee

**Affiliations:** 1 Neurophysiology Laboratory, Neurosurgical Service, Departments of Surgery, National Cheng Kung University Hospital, College of Medicine, National Cheng Kung University, Tainan 70403, Taiwan; 2 Department of Occupational Safety and Health, Chung Hwa University of Medical Technology, Tainan 71703, Taiwan

**Keywords:** stroke, melatonin, brain edema, aquaporin-4 (AQP4), metalloproteinase-9 (MMP-9)

## Abstract

**Background:** The efficacy of melatonin in reducing vasogenic and cytotoxic edema was investigated using a model of permanent middle cerebral artery occlusion (pMCAO). **Methods:** Rats underwent pMCAO, followed by intravenous administration of either melatonin (5 mg/kg) or a vehicle 10 min post-insult. Brain infarction and edema were assessed, and Western blot analyses were conducted to examine the expression levels of aquaporin-4 (AQP4), metalloproteinase-9 (MMP-9), and the neurovascular tight-junction protein ZO-1 upon sacrifice. The permeability of the blood–brain barrier (BBB) was measured using spectrophotometric quantification of Evans blue dye leakage. **Results:** Compared to controls, melatonin-treated rats exhibited a significant reduction in infarct volume by 26.9% and showed improved neurobehavioral outcomes (*p* < 0.05 for both). Melatonin treatment also led to decreased Evans blue dye extravasation and brain edema (*p* < 0.05 for both), along with lower expression levels of AQP4 and MMP-9 proteins and better preservation of ZO-1 protein (*p* < 0.05 for all). **Conclusions:** Therefore, melatonin offers neuroprotection against brain swelling induced by ischemia, possibly through its modulation of AQP4 and MMP-9 activities in glial cells and the extracellular matrix (ECM) during the early phase of ischemic injury.

## 1. Introduction

Ischemia is marked by excessive glutamate receptor activation and increased intracellular [Ca^2+^] (i) levels. This process not only heightens the vulnerability of neurons and glia to ischemic damage, causing cytotoxic edema, but also initiates damaging ischemic cascades at the blood–brain barrier (BBB) and extracellular matrix (ECM), resulting in increased vasogenic edema [1,2,3]. Thus, a key strategy for treating ischemic stroke patients involves reducing both cytotoxic and vasogenic edema during the acute phase, aiming to improve neurological outcomes by mitigating secondary brain damage due to severe brain edema [3,4].

Recent research highlights the crucial roles of the glial water channel protein aquaporin-4 (AQP4) and metalloproteinase-9 (MMP-9) activity in the ECM in maintaining brain water balance post-injury [4,5,6,7,8,9]. Studies on AQP4-deficient (AQP4−/−) mice have shown that AQP4 facilitates cytotoxic edema while reducing vasogenic edema following ischemic stroke [7,8,9]. In these AQP4−/− mice, the severity of acute brain injury after permanent focal cerebral ischemia is lessened due to reduced cytotoxic edema [8]. Additionally, AQP4 influences normal and post-injury astrocytic growth, migration, and scar formation, implicating it in the development of chronic brain injury and remodeling after an insult [10,11].

After cerebral ischemia, MMP-9 expression and activity are notably increased within 12–48 h post-insult [6,7]. Activated MMPs facilitate the entry of blood and inflammatory cells, as well as large, toxic molecules into the brain parenchyma, thereby exacerbating vasogenic hemorrhagic, transformation, brain edema, and neuronal damage post-insult [12,13,14].

Previous studies have demonstrated that melatonin (N-acetyl-5-methoxytryptamine) has various effects, effectively reducing acute brain swelling following ischemic stroke [5,6,14,15,16]. Melatonin and its metabolites are known for their potent free radical scavenging and antioxidant properties [17,18]. Melatonin is recognized for its ability to inhibit activated MMPs across various diseases [19,20,21,22,23]. We have previously shown that melatonin enhances the preservation of both early and late increases in blood–brain barrier permeability following ischemic stroke. [15,16]. Additionally, melatonin exerts its neuroprotective effects, in part, by modulating multiple pathways involved in inflammation and tissue damage. It regulates the plasminogen/plasmin system and endogenous MMP-9 inhibitors, leading to a reduction in systemic leukocyte migration and local microglial activation [5,6,24]. Moreover, melatonin potently inhibits MMP-9 activation by suppressing NF-κB signaling in LPS-stimulated RAW and BV2 cells [25].

While it is well established that melatonin protects against vasogenic edema resulting from BBB disruption following cerebral ischemia/reperfusion injuries, its potential to modulate post-insult AQP4 activation and thus mitigate cytotoxic edema after ischemic brain injury remains unclear. Further studies are needed to directly evaluate melatonin’s ability to inhibit cytotoxic edema and to elucidate its underlying mechanisms, as well as to examine its effects in vivo without the complication of reperfusion injury [25].

Here, we explored the neuroprotective effects of melatonin against vasogenic and cytotoxic edema following permanent focal cerebral ischemia in rats subjected, aiming to clarify the potential underlying mechanisms.

## 2. Materials and Methods

All procedures were conducted in accordance with the guidelines of the University’s Subcommittee on Research Animal Care (approval No. 105123). Chemicals were procured from Sigma-Aldrich Co., St. Louis, MO, USA, unless specified otherwise.

### 2.1. Animals

Adult male ICR mice weighing 30–35 g were sourced from the National Cheng Kung University Laboratory Animal Center. They had unrestricted access to food and water pre- and post-surgery. Animals were anesthetized with a gas mixture of 70% nitrous oxide and 30% oxygen, supplemented with 1–2% isoflurane. During surgery, a temperature-controlled heating pad (Harvard Apparatus, South Natick, MA, USA) was used to maintain core body temperature at 37 ± 0.5 °C.

### 2.2. Distal Middle Cerebral Artery Occlusion

Cauterizing the main distal branch of the right middle cerebral artery (pMCAO) using a modified Tamura model induced permanent focal cerebral ischemia [26,27]. A 10 mm vertical incision was made between the right eye and ear. A craniotomy was performed 2 mm rostral and 1 mm lateral to the foramen ovale. The dura mater was incised, and the right middle cerebral artery was coagulated and divided approximately 1 mm from the olfactory tract. The wound was sutured. Local cerebral blood flow (LCBF) was measured serially using laser-Doppler flowmetry (LDF, Laserflo BMP2, Vasamedics, St. Paul, MN, USA) before and 10 min after ischemia onset. LCBF data were expressed as a percentage of the baseline values in the study. Melatonin (Sigma-Aldrich Co.) was dissolved in a mixture of polyethylene glycol 400 (PEG 400, Sigma-Aldrich Co.) and 0.9% normal saline (3:7, *v*/*v*). Animals were randomly assigned, and the investigators were blinded to treatment paradigms. Animals received an intravenous injection of melatonin (5 mg/kg) or vehicle (PEG-saline) 10 min after ischemia onset. The dose of melatonin (5 mg/kg) was selected based on the dose–response relationship observed in previous studies using rodent stroke models [6,16].

### 2.3. Euthanasia and Quantification of Brain Infarct

Twenty-four hours post-surgery, animals were euthanized and perfused transcardially with 0.1 M phosphate-buffered saline followed by 4% paraformaldehyde. Brains were removed, embedded in optimal cutting temperature (OCT) compound (Miles Inc., Elkhart, IN, USA), and sectioned coronally at 40 μm using a cryostat (HM-500O, Mi-crom International GmbH, Waldorf, Germany). Serial sections were obtained from Bregma AP +2.22 to −4.78 mm at 1 mm intervals. Sections were mounted onto poly-L-lysine-coated slides (Sigma-Aldrich Co.) and dried overnight at 37 °C. Sections were stained with 0.5% cresyl violet to assess neu-ronal peri-nuclear ischemic injury.

Infarct areas were measured using a computerized image analyzer (MCID Elite; Imaging Research Inc., St. Catharines, ON, Canada), and infarct volumes were expressed as a percentage of the contralateral hemisphere volume [28,29,30]. Coronal sections from Bregma AP −0.22 to −0.78 mm were used to quantify surviving neurons. Three random, non-overlapping regions (500 × 400 µm²) were sampled from the ischemic core, inner, and outer boundary zones of infarction in the parietal cortex. Cell counts were expressed as the mean number of cells per mm².

### 2.4. Immunofluorescence Staining

Coronal brain sections were fixed in 4% paraformaldehyde for 5 min at room temperature and washed with PBS 3 times. The primary antibodies rabbit anti-aquaporin 4 monoclonal antibody (1:300, Epitomics, Inc., Burlingame, CA, USA) and mouse anti-glial fibrillary acidic protein (GFAP) monoclonal antibody (1:500, Ebiosceice, Inc., San Diego, CA, USA) were incubated with slides at 4 °C overnight [29,31]. A secondary antibody conjugated with biotin (1:100, Jackson ImmunoResearch, Inc., West Grove, PA, USA) was used, followed by incubation with FITC-conjugated streptavidin and Texas red-conjugated streptavidin (both 1:100, Jackson ImmunoResearch, Inc.). Pre-immune serum was used as a negative control, and no immunoreactivity was detected.

### 2.5. Evans Blue (EB) Leakage Assessment

Evans blue leakage was quantified on the melatonin-treated or control group by modifying previously reported methods [15,16]. Briefly, 0.15 mL of 1% Evans blue (Sigma-Aldrich Co.) dissolved in normal saline was injected intravenously under anesthesia 24 h post-ischemia. Each brain was weighed and homogenized in 150 μL of N,N-dimethyl formamide (Sigma-Aldrich Co.) after that, the homogenized was incubated for 72 h in a 50 °C water bath. Samples were centrifuged at 1500 g for 10 min. The supernatant was collected, and its absorbance was measured at 620 nm using a microplate reader (Stat Fax 2100, Awareness Technology, Inc., Palm. City, FL, USA). The quantity of Evans blue was determined by comparing the absorbance to a standard curve and was expressed as micrograms of Evans blue per gram of wet brain tissue.

### 2.6. Immunoblotting

Brain tissue was homogenized in lysis buffer containing 1% Triton X-100, 1% NP-40, 20 mM Tris-HCl (pH7.5), 1 mM EDTA, 1 mM EGTA, 1 μg/mL Leupeptin, 1 μg/mL Pepstatin, 1 μg/mL Aprotitin, 2 mM Na_3_VO_4_, 1 mM NaF, 1 mM Benzamidine, and 1 mM PMSF, and centrifuged at 18,000 g for 60 min at 4 °C. Protein concentrations were determined using a BCA protein assay kit (Pierce, Rockford, IL, USA). Sample protein (30 μg) was separated by 8–10% SDS-PAGE and transferred onto polyvinylidene difluoride membranes (IPVH00010; Millipore, Billerica, MA, USA). Membrane blocking was performed with 5% milk and processed with primary antibodies MMP-9 (monoclonal rabbit anti-MMP-9, 1:500; Chemicon International, Temecula, CA, USA), aquaporin4 (1:500; Santa Cruz Biotechnology, Inc., Santa Cruz, CA, USA), and ZO-1 (1:1000, Zymed, South San Francisco, CA, USA), followed by appropriate horseradish peroxidase-conjugated secondary antibodies (goat anti-rabbit or goat anti-mouse; 1:5000; Chemicon International) at room temperature for 1 h. Bound antibodies were visualized using the Amersham ECL system (RPN2132, GE Healthcare Bio-Sciences Corp., Piscataway, NJ, USA). Membranes were then probed for β-actin (1:10,000; Chemicon International). To determine the specificity of the primary antibodies, pre-absorbed antibodies with blocking peptides were used instead of primary antibodies. Optical densities were measured using a Luminescent Image Analyzer (Fujifilm LAS-3000; Fuji Photo Film Co., Tokyo, Japan).

### 2.7. Electromicroscopic Examinations

Animals were perfused with 1% glutaraldehyde in PBS (PBS; Molecular Probes, Eugene, OR, USA). Brains were then post-fixed in the same fixative for 24 h. After rinsing three times in distilled water for 5 min each, tissues were post-fixed in 2% osmium tetroxide (OsO4) until black. Tissues were then dehydrated in a graded series of ethanol (75%, 85%, 95% for 5 min each, and 100% for 15 min each). After dehydration, tissues were infiltrated with propylene oxide for 10 min and then embedded in a 1:1 mixture of propylene oxide and epoxy resin for 24 h. Subsequently, tissues were transferred to pure epoxy resin and placed under vacuum for 4–5 h. Tissues were then sandwiched between two gelatin capsules and polymerized overnight at 65–75 °C. Polymerized blocks were sectioned using an ultramicrotome. Ultrathin sections were collected on formvar-coated nickel grids. Non-specific binding was blocked with saturated sodium metaperiodate. After washing extensively with distilled water, sections were incubated with the primary antibody for 2 h at 37 °C. Following extensive washing with PBS, sections were incubated with the secondary gold-conjugated antibody for 2 h. After additional washing with PBS and distilled water, sections were stained with uranyl acetate and lead citrate and then air-dried. Ultrathin sections were cut and visualized using a transmission electron microscope (JEM-1400, JEOL Ltd., Tokyo, Japan).

### 2.8. Neurobehavioral Scoring

Body weight was monitored before the ischemic brain insult and euthanasia. Two observers who were unaware of the treatment protocol conducted two well-established neurologic grading systems before the ischemic brain insult and euthanasia. A sensorimotor grading scale modified from previously published methods was used [28,32,33]. The affected forelimb underwent forward and sideways visual placing tests, which were scored as follows: 0, complete immediate placement; 1, incomplete and/or delayed placement (<2 s); and 2, absence of placement. The five categories of motor neurologic findings were scored as follows: 0, no observable deficit; 1, forelimb flexion; 2, forelimb flexion and decreased resistance to lateral push; 3, forelimb flexion, decreased resistance to lateral push and unilateral circling; and 4, forelimb flexion, unable or difficult to ambulate. Animals were also rated using a scale developed by Clark et al., with scores ranging 0–28 [34]. Higher scores indicate more severe motor function impairment caused by ischemic brain injury.

### 2.9. Statistical Analysis

Data are presented as mean ± SD. To assess the response to changing conditions, a paired Student’s t-test was applied. For group comparisons, an unpaired Student’s t-test was used for between-group comparisons, while one-way analysis of variance (ANOVA) followed by Fisher’s least significant difference (LSD) post hoc test was used for multiple group comparisons. For neurobehavioral scores, the Kruskal–Wallis test and Mann–Whitney U test were employed. Statistical significance was defined as *p* < 0.05.

## 3. Results

**Figure 1:**
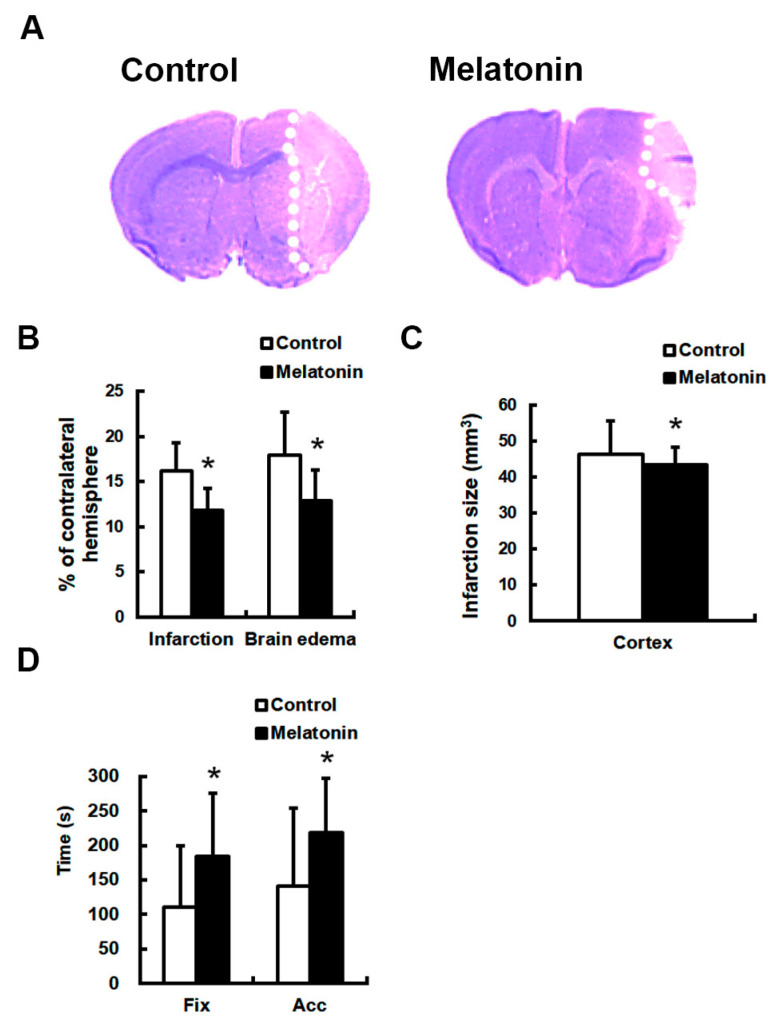
Melatonin treatment significantly reduced infarct volume and improved neurological outcomes in mice subjected to permanent focal cerebral ischemia (pMCAO). Animals were randomly assigned to receive either PEG 400-saline (*n* = 13) or melatonin (5 mg/kg, *n* = 12) following pMCAO. Cresyl violet staining revealed substantially smaller infarct areas in the melatonin-treated group compared to controls (**A**,**B**). Moreover, melatonin treatment significantly increased the number of surviving neurons in the penumbra (**C**), leading to improved motor function as assessed by the Rota rod test (**D**) (* *p* < 0.05 vs. controls).

Five animals died before the completion of the experimental procedures following pMCAO and were excluded from the study. Two were from the vehicle group and three were from the melatonin treatment group. After ischemic onset, the ipsilateral LCBF declined to 11.2–20.4% of baseline levels in the ischemic territory. LCBF levels were not significantly different between vehicle-injected and melatonin-treated animals and were unaffected by melatonin treatment (*p* > 0.05). Compared to controls (*n* = 13), animals treated with melatonin (5 mg/kg; *n* = 12) exhibited significant reductions in infarct volume by 26.9% and brain edema by 28.3% (*p* < 0.05 for both; Figure 1A,B), and a 31.5% increase in the number of surviving cells in penumbral regions (*p* < 0.05; Figure 1C). Melatonin treatment also significantly improved motor and 28-point clinical scale neurobehavioral outcomes (*p* < 0.05; Table 1). Additionally, melatonin-treated mice showed significant improvements in poststroke motor performance, both in the fixed and accelerated modes, as determined by Rota-rod testing (*p* < 0.05; Figure 1D).

**Figure 2:**
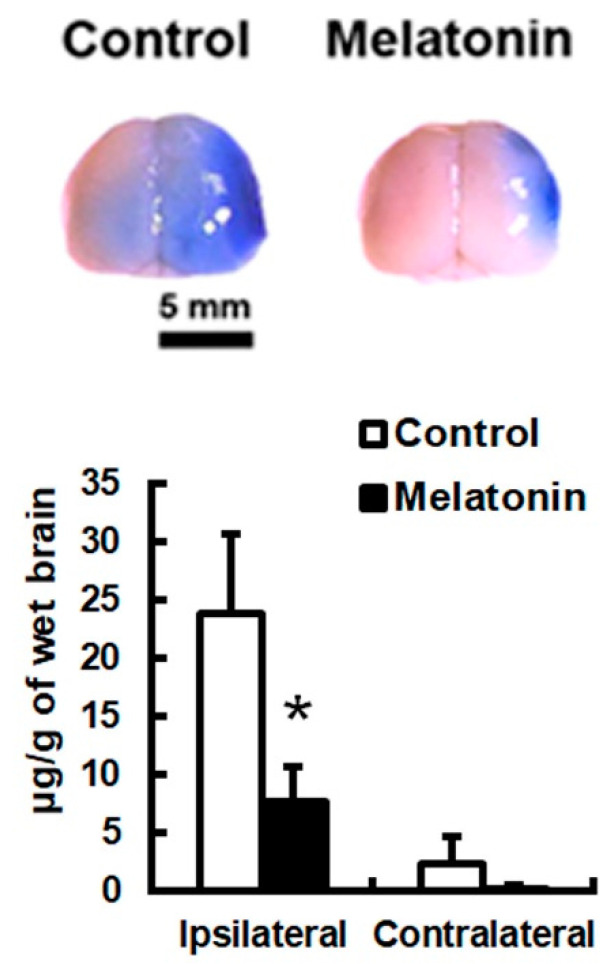
Melatonin treatment reduced blood–brain barrier (BBB) permeability following permanent focal cerebral ischemia. Evans blue extravasation was significantly lower in melatonin-treated animals compared to controls (* *p* < 0.05, *n* = 6). Scale bar = 5 mm.

Cerebral ischemia consistently increased BBB permeability and brain edema in the ipsilateral (ischemic) hemisphere. Animals that received melatonin (5 mg/kg) 10 min after proximal MCA occlusion showed a significant reduction in Evans blue extravasation in the ipsilateral (ischemic) hemisphere by 67.4% at 24 h post-insult (*p* < 0.05; *n* = 6; Figure 2), but not in the contralateral (non-ischemic) hemisphere, compared to vehicle (PEG-saline)-injected controls.

**Figure 3:**
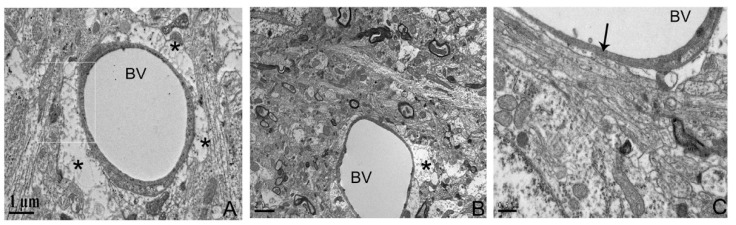
Melatonin attenuated the rises in the brain edema by reducing the swelling of astrocytic processes around the blood vessels in the penumbra. Transmission electron microscopy images of the penumbra region in the ischemic hemisphere. The images show the penumbra region of the ipsilateral (ischemic) hemisphere. (**A**) In the vehicle control group, perivascular astrocytes exhibit edema (asterisk). (**B**) In the melatonin-treated group, perivascular astrocyte edema (asterisk) is reduced compared to the control group. The structural integrity of the blood–brain barrier (arrow) can be observed after melatonin administration (**C**). Scale bar = 1 μM (**A**), 2 μM (**B**), and 0.5 μM (**C**).

**Figure 4:**
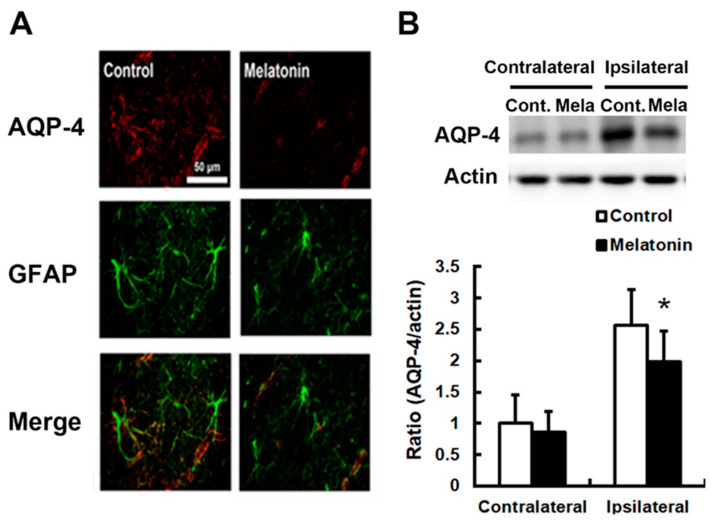
Melatonin attenuated the expression of aquaporin-4 (AQP4) following permanent focal cerebral ischemia. (**A**) Immunohistochemistry revealed that the AQP4 was significantly reduced by melatonin treatment following permanent focal cerebral ischemia after 24 h. (**B**) The melatonin-mediated brain edema reduction was accompanied by reduced levels in the APQ4. Scale bar = 50 μM. (* *p* < 0.05 vs. controls, and *n* = 9).

**Figure 5:**
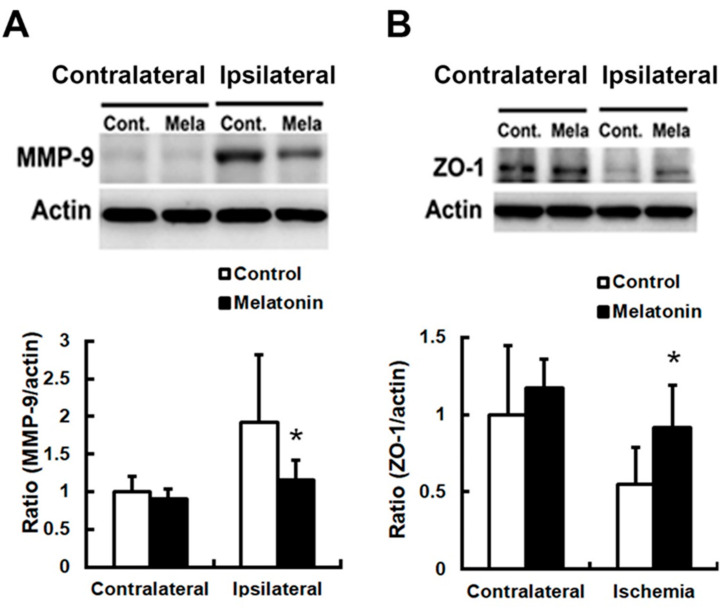
Melatonin attenuated the increased expression of metalloproteinase-9 (MMP-9) and ZO-1 following permanent focal cerebral ischemia. (**A**) Melatonin-treated animals had significantly reduced MMP-9 expression compared to controls. Consequently, melatonin treatment improved (**B**) the preservation of the ZO-1 protein. (* *p* < 0.05 vs. controls, and *n* = 9).

Post-ischemic increases in BBB permeability and brain edema were associated with increased expressions of AQP4 and MMP-9. We observed reduced swelling of astrocytic processes around blood vessels in the penumbra of the melatonin-treated group (Figure 3). The decrease in brain edema induced by melatonin was accompanied by reduced AQP4 levels by 22.8%, compared to control values (*p* < 0.05; *n* = 9; Figure 4A,B). Additionally, melatonin-treated animals showed significantly reduced MMP-9 expression by 39.6% (*p* < 0.05; *n* = 9; Figure 5A) and better preservation of ZO-1 protein by 67.4% (*p* < 0.05; *n* = 9; Figure 5B).

## 4. Discussion

Our study demonstrated that melatonin (5 mg/kg) significantly reduced postischemic increases in BBB permeability, brain edema, and the elevated expressions of MMP9 and AQP4 proteins following permanent focal cerebral ischemia in mice. Additionally, our findings indicated that melatonin treatment significantly preserved the ZO-1 protein and the cytoarchitecture of the neurovascular unit. Consequently, melatonin-treated animals exhibited significantly reduced infarct volumes, improved survival of neurons in the penumbral areas, and superior neurobehavioral outcomes following permanent focal cerebral ischemia. This neuroprotection in reducing postischemic brain edema and preserving the neurovascular unit cannot be attributed to differences in mean arterial blood pressure, as these were not significantly different between vehicle-injected and melatonin-treated animals.

We demonstrated that melatonin effectively reduced post-injury activations of AQP4 and associated cytotoxic edema following permanent focal cerebral ischemia. It is likely that other mechanisms of melatonin, such as its ability to improve post-injury intracellular calcium inflow [Ca^2+^] (i) or reduce pH, may also contribute independently or in combination with its inhibition of AQP4 activation to attenuate post-injury cytotoxic edema in vitro and in vivo [1,2,3,35].

MMP-9 activation plays a significant role in the pathogenesis of a wide range of central nervous system disorders, such as ischemic stroke, meningitis, and encephalitis [20,21,22,23,25]. We have previously demonstrated in neuronal cell cultures and animal models that melatonin-mediated inhibition of MMP-9 reduces local brain injury [5,6,25]. In the present study, we showed that melatonin protected against post-ischemic MMP-9 activation following permanent focal cerebral ischemia in mice. Therefore, melatonin exhibits a cross-species, multi-potent inhibitory effect on both systemic and local MMP-9 activation following various central nervous system injuries, targeting different organs [20,21,22,23,25].

Furthermore, excessive glutamate release after ischemic stroke induces excitotoxicity, resulting in brain damage and edema. Previous studies have reported that melatonin activates NMDAR-containing NR2a subunits in pMCAO animal models, leading to the activation of pro-survival signaling [36]. Nevertheless, the precise molecular mechanisms underlying melatonin’s neuroprotective effects require further investigation.

Previous studies have indicated that sleep disruption exacerbates the risk of increased hypertension, vasospasm, and blood–brain barrier (BBB) disruption in patients with traumatic brain injury (TBI), while also affecting neuroplasticity [37]. In addition, hydrocephalus following subarachnoid hemorrhage (SAH) is caused by AQP4 dysfunction, lymphatic obstruction, and vasospasm. Melatonin’s regulation of AQP4 and its protective effects on the BBB present a potential therapeutic approach [38].

These findings strongly suggest that melatonin could be a promising therapeutic agent for directly reducing post-ischemic brain edema, and could also serve as an adjunctive therapy for thrombolytic treatment of ischemic stroke [15,16,24]. While melatonin shows promise for treating ischemic stroke patients who present outside the thrombolytic window, additional clinical trials are necessary to establish its efficacy and safety [39].

## 5. Conclusions

Our results demonstrated that melatonin effectively inhibits post-injury increases in AQP4 activation and cytotoxic edema in vivo, as well as cerebral MMP-9 activation and vasogenic edema following permanent focal cerebral ischemia. These findings provide direct evidence of melatonin’s pluripotent mechanisms for attenuating postinsult cytotoxic and vasogenic edema, supporting its use either directly to reduce postischemic brain edema or as an adjunct to thrombolytic therapy for ischemic stroke patients.

## Figures and Tables

**Table 1:** Melatonin improves sensorimotor behavioral scores after permanent focal cerebral ischemia in mice.

	Neurologic Behavioral Score		
	Sensory	Motor	28-Point Clinical Scale
Vehicle-treated (*n* = 13)	2.0 (1.60–2.40)	2.5 (1.99–3.01)	12.5 (9.95–15.05)
Melatonin-treated (*n* = 12)	1.0 (0.55–1.45)	1.0 (0.9–1.1) *	7.5 (6.64–8.36) *

Neurologic behavioral scores are expressed as medians (95% CI). * *p* < 0.05 vs. vehicle data, respectively.

## Data Availability

The data that support the findings of this study are available from the corresponding author upon reasonable request.

## References

[1] Choi D.W. (1988). Glutamate neurotoxicity and diseases of the nervous system. Neuron.

[2] Hazell A.S. (2007). Excitotoxic mechanisms in stroke: An update of concepts and treatment strategies. Neurochem. Int..

[3] Zhao Y., Dou J., Luo J., Li W., Chan H.H., Cui W., Zhang H., Han R., Carlier P.R., Zhang X. (2011). Neuroprotection against excitotoxic and ischemic insults by bis(12)-hupyridone, a novel anti-acetylcholinesterase dimer, possibly via acting on multiple targets. Brain Res..

[4] Gasche Y., Copin J.C., Sugawara T., Fujimura M., Chan P.H. (2001). Matrix metalloproteinase inhibition prevents oxidative stress-associated blood-brain barrier disruption after transient focal cerebral ischemia. J. Cereb. Blood Flow. Metab..

[5] Hung Y.C., Chen T.Y., Lee E.J., Chen W.L., Huang S.Y., Lee W.T., Lee M.Y., Chen H.Y., Wu T.S. (2008). Melatonin decreases matrix metalloproteinase-9 activation and expression and attenuates reperfusion-induced hemorrhage following transient focal cerebral ischemia in rats. J. Pineal Res..

[6] Tai S.H., Chen H.Y., Lee E.J., Chen T.Y., Lin H.W., Hung Y.C., Huang S.Y., Chen Y.H., Lee W.T., Wu T.S. (2010). Melatonin inhibits postischemic matrix metalloproteinase-9 (MMP-9) activation via dual modulation of plasminogen/plasmin system and endogenous MMP inhibitor in mice subjected to transient focal cerebral ischemia. J. Pineal Res..

[7] Yang B., Zador Z., Verkman A.S. (2008). Glial cell aquaporin-4 overexpression in transgenic mice accelerates cytotoxic brain swelling. J. Biol. Chem..

[8] Manley G.T., Fujimura M., Ma T., Noshita N., Filiz F., Bollen A.W., Chan P., Verkman A.S. (2000). Aquaporin-4 deletion in mice reduces brain edema after acute water intoxication and ischemic stroke. Nat. Med..

[9] Papadopoulos M.C., Manley G.T., Krishna S., Verkman A.S. (2004). Aquaporin-4 facilitates reabsorption of excess fluid in vasogenic brain edema. Faseb J..

[10] Saadoun S., Papadopoulos M.C., Watanabe H., Yan D., Manley G.T., Verkman A.S. (2005). Involvement of aquaporin-4 in astroglial cell migration and glial scar formation. J. Cell Sci..

[11] Verkman A.S., Binder D.K., Bloch O., Auguste K., Papadopoulos M.C. (2006). Three distinct roles of aquaporin-4 in brain function revealed by knockout mice. Biochim. Biophys. Acta.

[12] Zhao B.Q., Ikeda Y., Ihara H., Urano T., Fan W., Mikawa S., Suzuki Y., Kondo K., Sato K., Nagai N. (2004). Essential role of endogenous tissue plasminogen activator through matrix metalloproteinase 9 induction and expression on heparin-produced cerebral hemorrhage after cerebral ischemia in mice. Blood.

[13] Romanic A.M., White R.F., Arleth A.J., Ohlstein E.H., Barone F.C. (1998). Matrix metalloproteinase expression increases after cerebral focal ischemia in rats: Inhibition of matrix metalloproteinase-9 reduces infarct size. Stroke.

[14] Torii K., Uneyama H., Nishino H., Kondoh T. (2004). Melatonin suppresses cerebral edema caused by middle cerebral artery occlusion/reperfusion in rats assessed by magnetic resonance imaging. J. Pineal Res..

[15] Chen T.Y., Lee M.Y., Chen H.Y., Kuo Y.L., Lin S.C., Wu T.S., Lee E.J. (2006). Melatonin attenuates the postischemic increase in blood-brain barrier permeability and decreases hemorrhagic transformation of tissue-plasminogen activator therapy following ischemic stroke in mice. J. Pineal Res..

[16] Chen H.Y., Chen T.Y., Lee M.Y., Chen S.T., Hsu Y.S., Kuo Y.L., Chang G.L., Wu T.S., Lee E.J. (2006). Melatonin decreases neurovascular oxidative/nitrosative damage and protects against early increases in the blood-brain barrier permeability after transient focal cerebral ischemia in mice. J. Pineal Res..

[17] Tan D.X., Manchester L.C., Terron M.P., Flores L.J., Reiter R.J. (2007). One molecule, many derivatives: A never-ending interaction of melatonin with reactive oxygen and nitrogen species?. J. Pineal Res..

[18] Hardeland R., Tan D.X., Reiter R.J. (2009). Kynuramines, metabolites of melatonin and other indoles: The resurrection of an almost forgotten class of biogenic amines. J. Pineal Res..

[19] Esposito E., Genovese T., Caminiti R., Bramanti P., Meli R., Cuzzocrea S. (2008). Melatonin regulates matrix metalloproteinases after traumatic experimental spinal cord injury. J. Pineal Res..

[20] Paul S., Sharma A.V., Mahapatra P.D., Bhattacharya P., Reiter R.J., Swarnakar S. (2008). Role of melatonin in regulating matrix metalloproteinase-9 via tissue inhibitors of metalloproteinase-1 during protection against endometriosis. J. Pineal Res..

[21] Esposito E., Mazzon E., Riccardi L., Caminiti R., Meli R., Cuzzocrea S. (2008). Matrix metalloproteinase-9 and metalloproteinase-2 activity and expression is reduced by melatonin during experimental colitis. J. Pineal Res..

[22] Ganguly K., Swarnakar S. (2009). Induction of matrix metalloproteinase-9 and -3 in nonsteroidal anti-inflammatory drug-induced acute gastric ulcers in mice: Regulation by melatonin. J. Pineal Res..

[23] Mishra A., Paul S., Swarnakar S. (2011). Downregulation of matrix metalloproteinase-9 by melatonin during prevention of alcohol-induced liver injury in mice. Biochimie.

[24] Lee M.Y., Kuan Y.H., Chen H.Y., Chen T.Y., Chen S.T., Huang C.C., Yang I.P., Hsu Y.S., Wu T.S., Lee E.J. (2007). Intravenous administration of melatonin reduces the intracerebral cellular inflammatory response following transient focal cerebral ischemia in rats. J. Pineal Res..

[25] Chang C.C., Tien C.H., Lee E.J., Juan W.S., Chen Y.H., Hung Y.C., Chen T.Y., Chen H.Y., Wu T.S. (2012). Melatonin inhibits matrix metalloproteinase-9 (MMP-9) activation in the lipopolysaccharide (LPS)-stimulated RAW 264.7 and BV2 cells and a mouse model of meningitis. J. Pineal Res..

[26] Tamura A., Graham D.I., McCulloch J., Teasdale G.M. (1981). Focal cerebral ischaemia in the rat: 1. Description of technique and early neuropathological consequences following middle cerebral artery occlusion. J. Cereb. Blood Flow. Metab..

[27] Hung Y.C., Chou Y.S., Chang C.H., Lin H.W., Chen H.Y., Chen T.Y., Tai S.H., Lee E.J. (2010). Early reperfusion improves the recovery of contralateral electrophysiological diaschisis following focal cerebral ischemia in rats. Neurol. Res..

[28] Lee E.J., Lee M.Y., Chen H.Y., Hsu Y.S., Wu T.S., Chen S.T., Chang G.L. (2005). Melatonin attenuates gray and white matter damage in a mouse model of transient focal cerebral ischemia. J. Pineal Res..

[29] Lee E.J., Chen H.Y., Lee M.Y., Chen T.Y., Hsu Y.S., Hu Y.L., Chang G.L., Wu T.S. (2005). Cinnamophilin reduces oxidative damage and protects against transient focal cerebral ischemia in mice. Free Radic. Biol. Med..

[30] Swanson R.A., Morton M.T., Tsao-Wu G., Savalos R.A., Davidson C., Sharp F.R. (1990). A semiautomated method for measuring brain infarct volume. J. Cereb. Blood Flow. Metab..

[31] Chen T.Y., Tai S.H., Lee E.J., Huang C.C., Lee A.C., Huang S.Y., Wu T.S. (2011). Cinnamophilin offers prolonged neuroprotection against gray and white matter damage and improves functional and electrophysiological outcomes after transient focal cerebral ischemia. Crit. Care Med..

[32] Chen T.Y., Lin M.H., Lee W.T., Huang S.Y., Chen Y.H., Lee A.C., Lin H.W., Lee E.J. (2012). Nicotinamide inhibits nuclear factor-kappa B translocation after transient focal cerebral ischemia. Crit. Care Med..

[33] Belayev L., Alonso O.F., Busto R., Zhao W., Ginsberg M.D. (1996). Middle cerebral artery occlusion in the rat by intraluminal suture. Neurological and pathological evaluation of an improved model. Stroke.

[34] Clark W.M., Rinker L.G., Lessov N.S., Hazel K., Hill J.K., Stenzel-Poore M., Eckenstein F. (2000). Lack of interleukin-6 expression is not protective against focal central nervous system ischemia. Stroke.

[35] Slivka A., Murphy E., Horrocks L. (1995). Cerebral edema after temporary and permanent middle cerebral artery occlusion in the rat. Stroke.

[36] Shah F.A., Liu G., Al Kury L.T., Zeb A., Abbas M., Li T., Yang X., Liu F., Jiang Y., Li S. (2019). Melatonin Protects MCAO-Induced Neuronal Loss via NR2A Mediated Prosurvival Pathways. Front. Pharmacol..

[37] Lucke-Wold B.P., Smith K.E., Nguyen L., Turner R.C., Logsdon A.F., Jackson G.J., Huber J.D., Rosen C.L., Miller D.B. (2015). Sleep disruption and the sequelae associated with traumatic brain injury. Neurosci. Biobehav. Rev..

[38] Motwani K., Dodd W.S., Laurent D., Lucke-Wold B., Chalouhi N. (2022). Delayed cerebral ischemia: A look at the role of endothelial dysfunction, emerging endovascular management, and glymphatic clearance. Clin. Neurol. Neurosurg..

[39] Sánchez-Barceló E.J., Mediavilla M.D., Tan D.X., Reiter R.J. (2010). Clinical uses of melatonin: Evaluation of human trials. Curr. Med. Chem..

